# Burdens and coping strategies among geriatric care nurses during COVID-19: A factorial survey

**DOI:** 10.1016/j.ijnsa.2025.100386

**Published:** 2025-07-19

**Authors:** Christopher Huken, Patrick Kutschar, Martin W. Schnell, Christine Dunger

**Affiliations:** a Chair of Social Philosophy and Ethics in Health Care, Witten/Herdecke University, Germany; b Institute of Nursing Science and Practice, Paracelsus Medical University, Salzburg, Austria; c Center for Public Health and Healthcare Research, Paracelsus Medical University, Salzburg, Austria

**Keywords:** Geriatric nursing, Caregiver burden, Coping strategies, Germany, COVID-19, Factorial survey, Multilevel analysis

## Abstract

**Background:**

Geriatric care nurses used different strategies to cope with the various burdens they faced during the COVID-19 pandemic, affecting care interactions and reflecting their professional identity.

**Objective:**

To explore the burdens and coping strategies of German geriatric care nurses, using professional identity as a conceptual framework.

**Design:**

Cross-sectional factorial survey using vignettes (i.e., hypothetical descriptions of care situations).

**Setting & Participants:**

Stratified sampling was applied at the facility level, based on the distribution of nursing homes across German federal states. A total of 188 geriatric care nurses participated.

**Methods:**

The factorial online survey included socio-demographic variables, COVID-19-specific questions, vignettes, and feedback items. Descriptive statistics and multilevel regression analysis were conducted.

**Results:**

Participants reported higher stress during the pandemic than before. Burden characteristics described in the vignettes had no significant effect on participants' judgement behaviour. A random-intercept level 2 model showed that a higher workload and caring for residents significantly influenced participants' judgement. Closeness to family and friends, and seeking support from colleagues were effective coping strategies, while bending guidelines and harmful consumption behaviour were less effective.

**Conclusions:**

Coping strategies focused on social interaction were particularly beneficial despite contact restrictions. The type of burden appeared less important than the ability to cope with it. The subordinate role of professional identity may reflect a shift towards functional care. We have highlighted the possible need to address stress management and working conditions of geriatric care nurses, especially in the light of potential future crises.

**Registration:**

None

**Tweetable abstract:**

A factorial survey exploring relevant burden and coping strategies German geriatric care nurses experienced during the COVID-19 pandemic.

**What is already known**

• Many nurses experienced increased stress during the COVID-19 pandemic and faced various challenges that they had to overcome.
• There was a particularly high number of COVID-19-related infections and deaths in geriatric care.
• In an international comparison, only a few researchers focused on the stress experience and coping strategies of geriatric care nurses in nursing homes.

Alt-text: Unlabelled box

**What this paper adds**

• We have connected burdens and coping strategies with each other in the light of professional identity.
• We have shown how geriatric care nurses rated certain COVID-19 associated burdens and coping strategies.
• Seeking social support from friends, family members, and colleagues was shown to be a reliable coping strategy in times of crisis.

Alt-text: Unlabelled box

## 1 Background

On 21 April 2024, the World Health Organization stated that over 775 million coronavirus disease 2019 (COVID-19) cases had been reported (World Health Organization, 2023). In an international comparison, Germany ranked fifth with 38.4 million reported COVID-19 cases. The Robert Koch Institute recorded a total of 2338,915 COVID-19 cases in the entire German population between March 2020 and February 2021 (Schweickert et al., 2021). Of these, 18.9 % were over the age of 65. Looking at the outbreaks in German nursing homes, the Robert Koch Institute reported that a total of 132,952 outbreaks were registered in the period described and that one in five outbreaks occurred in people over the age of 65 in a nursing home. Almost 50 % of COVID-19 outbreaks in German nursing homes were accompanied by deaths and often led to severe courses of the disease (Schweickert et al., 2021). The burden experienced by geriatric care nurses, which was already tense before the COVID-19 pandemic (Jacobs et al., 2020), was exacerbated by the new pandemic challenges (Hoedl et al., 2022).

### 1.1 COVID-19 pandemic-related burdens

The negative psychological and physical impact of the COVID-19 pandemic on the health of residents was particularly difficult for many geriatric care nurses. As a result, many felt they were no longer able to meet the social and emotional needs of residents (Eich-Krohm et al., 2022). This was exacerbated by disputes with relatives who wanted to visit their family members but often did not adhere to the hygiene and safety regulations (Müller et al., 2021). Further burdens were compliance with and implementation of the new hygiene and protection requirements (Diehl et al., 2022; Schulze et al., 2022), the need for non-infected geriatric care nurses to compensate for the absence of colleagues who had contracted COVID-19 (Müller et al., 2021), and the wearing of personal protective equipment (Hoedl et al., 2022).

In addition, many of the geriatric care nurses were afraid of infecting themselves or others with the COVID-19 virus (Begerow and Gaidys, 2022; Diehl et al., 2022). In the CoViRiS study, Rosner et al. (2024) showed that participants who worked in the healthcare sector were more likely to have a symptomatic COVID-19 infection. The fear of a possible infection was also associated with negative experiences with the COVID-19 antigen tests (Schulze et al., 2022). Due to the changed working conditions as a result of the COVID-19 hygiene and protection requirements, geriatric care nurses were confronted with new burdens in their everyday work. Looking at the psychological consequences of stress, depression and anxiety were associated with COVID-19-related stress (Hering et al., 2022).

### 1.2 COVID-19 pandemic-related coping strategies

Geriatric care nurses responded to the burdens with different coping strategies. Interpersonal dialogue with colleagues, family members, and residents was listed in various publications as one of the most effective coping strategies (Diehl et al., 2022; Eich-Krohm et al., 2022; Hoedl et al., 2022). This included, for example, carers asking staff from other areas of the facility for help (Diehl et al., 2022). To cope with the increased workload, many of the geriatric care nurses adapted their working habits. This adaptation manifested itself in a more flexible way of interacting with residents (Diehl et al., 2022) or in autonomously deciding whether a resident could receive visitors (Flatscher-Thöni et al., 2022). To minimize the spread of the COVID-19 virus in the facility – but also in the private environment – many geriatric care nurses restricted their social contacts with residents, as well as with family and friends (Müller et al., 2021; Schulze et al., 2022).

### 1.3 Professional identity during the COVID-19 pandemic

The diverse burdens of the COVID-19 pandemic affected not only the coping strategies of geriatric care nurses but also their professional identity. There is no clear definition of professional identity in the literature. However, authors generally refer to self-concept (Johnson et al., 2012). Sutherland et al. (2010) emphasise professional identity as a component of the multiple perspectives of identity. They define that a person's professional identity “comes from his/her position within society, his/her interactions with others and his/her interpretations of his/her experiences” (Sutherland et al., 2010, p. 455). Because professional identity is one of the dimensions of a person's own identity or self-concept (Berg, 2017), every geriatric care nurse has a certain idea of what it means to be and how to act as a good nurse (Fagermoen, 1997). This idea is individually different and flexible (Rasmussen et al., 2018) and is one of the main components of professional identity.

In the study by Philippa et al. (2021), nurses described “[…] that critical thinking and problem solving with confidence, empathy and autonomy were necessary to provide safe, effective and evidence-based care” (p. 5). At the same time, the underlying needs for autonomy and competence – as formulated in Deci and Ryan's (2000) self-determination theory – could often not be met during the pandemic. The freedom of action of geriatric care nurses was restricted and their understanding of care was shaken by a loss of authenticity and professionalism (Begerow and Gaidys, 2022). Due to the isolation measures and the resulting change in proximity to the residents, many geriatric care nurses felt that they could no longer meet their relational function as carers (Flatscher-Thöni et al., 2022). The fit between the professional identity constructed by the geriatric care nurses and the actual working environment was incongruent, which placed an additional burden on the geriatric care nurses and required them to develop coping strategies. In addition, many carers were sceptical about the heroisation of their profession as a result of the pandemic (Diehl et al., 2022; Hennekam et al., 2020).

To date, there is little research that explicitly examines the coping strategies that geriatric care nurses chose in response to the burdens caused by the pandemic, considering their professional identity. We examined the following question:

*Which coping strategies were chosen by geriatric care nurses in nursing home care considering their professional identity under the special stresses of the COVID-19, and which proved themselves from the participants' perspectives?*

## 2 Methods

The factorial survey was part of a mixed-method project on the burdens and coping strategies of geriatric care nurses during the COVID-19 pandemic ("Professional Identity and Coping Strategies of Nurses in the Face of the Corona Crisis"). The project, funded by the German Federal Ministry of Education and Research (funding code: 01UP2223), includes three phases: the present cross-sectional factorial survey, the development of scenario-based recommendations using the nominal group technique, and a final phase that is examining public discourse on geriatric nursing and will propose recommendations.

### 2.1 Study design and participants

In this cross-sectional factorial survey, we focused on the geriatric care nurse’s assessment of vignettes. Vignettes are fictional but realistic text descriptions of people or situations in which individual text modules are systematically varied, such as personal (e.g., sex or age) or situational factors (e.g., high infection risk). These factors are called ‘dimensions’. The levels of the dimensions are known as ‘characteristics’. For example, the characteristics of the dimension sex could be ‘male’ or ‘female’. In the literature review process, researchers identify the dimensions and characteristics relevant to their research work and implement them in a text framework. By varying the characteristics, their individual influence on the participants' judgement behaviour can be determined and compared.

The sample was derived from the population of geriatric care nurses working in German nursing homes. In Germany, a geriatric care nurse is a state-recognised and certified nurse who has completed training or studies. Most German geriatric care nurses have generally completed a special 2- to 3-year vocational training program in geriatric care. Further inclusion criteria were that

• the participants had more than 2 years of work experience, meaning they were active in geriatric nursing care during the COVID-19 pandemic (2020 – 2023),
• worked in a nursing home, and
• were fluent in German.

Stratified random sampling at the facility (i.e., nursing home) level was applied. In this process, the percentage distribution of nursing homes in the respective federal states was determined based on the long-term care facility address list of the BIVA (Bundesinteressenvertretung für alte und pflegebetroffene Menschen e.V.), a federal advocacy group in Germany. Facilities that mainly provided geriatric care were contacted. Day care facilities or facilities that mainly cared for residents with geriatric psychiatric disorders were excluded. Of the total of 15,024 contact addresses, 2600 eligible nursing homes were randomly selected. The nursing home managers were contacted in the form of an e-mail cover letter containing an information and clarification letter, as well as an invitation to the study, that the nursing home managers could forward to the relevant geriatric care nurses. In addition to the initial contact, two reminders were sent out to encourage participation in the study. A total of 3423 reminders were sent out.

The sampling procedure was continued until the a priori determined sample size of *N*= 192 was met. The sample size was calculated based on the recommendation of 12 judgements per vignette (Beck and Opp, 2001). As the total of 96 vignettes were divided into 16 sets, this results in a calculation of 12 × 16 = 192 participants.

### 2.2 Measures

The factorial survey consisted of a socio-demographic questionnaire section, a section containing selected COVID-19-specific variables, and the vignettes. As a basis for questionnaire and vignette construction, a literature search was carried out at the beginning to identify the burdens and coping strategies relevant for the creation of the vignettes. The factorial survey was then pre-tested by nine nursing and methodological experts. The feedback from these experts was integrated into the final version of the questionnaire. The online factorial survey was made available to the participants via LimeSurvey (LimeSurvey GmbH, n. d.) between 8 August 2023 and 9 January 2024. The survey duration was anticipated to be 15 to 20 min, and the questionnaire contained five online screen pages. Backward navigation was not permitted. However, participants had the option of saving their progress and continuing later. They were also free to skip questions. Before the survey began, the participants were informed about the conditions of the data and privacy policies on the landing page. The information and clarification letter were also available for them to download.

#### 2.2.1 Sociodemographic and COVID-19-specific variables

The socio-demographic questionnaire section (Supplementary Material, Table S1) was presented to the participants at the beginning of the factorial survey, including personal data, such as gender, age, highest qualification, and personal circumstances of the participants, as well as facility-specific data (federal state in which the participants worked, the type of organisation, the number of nursing home beds). Further variables were collected that recorded the professional situation of the participants (nursing experience, direct nursing care for residents [i.e., providing hands-on care to residents], the amount of working time during the pandemic).

In addition to the personal and facility-related variables, potentially burdensome variables of the COVID-19 pandemic were included (e.g., working overtime, infection events from participants’ direct environment, estimated number of infected residents, deaths experienced because of an infection).

Nurses’ experience of stress, personal resources, perceived experience of competence and autonomy, consumption of substances, and recreational consumption were presented as formulated statements in a matrix-grid format and agreement was measured using 5-point Likert-type scales.

Next to feedback on the comprehensibility of the vignettes, open-format text fields were to report aspects that were not covered by the factorial survey but were particularly relevant to the participants.

#### 2.2.2 Vignettes

The burdens and the coping strategies identified in the literature were finalised by consensus within the research team and summarised in a vignette universe. The vignettes described the burdens and coping strategies of a fictional geriatric care nurse during the COVID-19 pandemic and were presented to the participants. The vignette universe included *N*= 96 vignettes after combining all possible characteristics (Table 1). The characteristics were combined using the 'ExcelKutools' program (ExtendOffice Technology Inc., 2008). The dimensions 'COVID-19 infection', 'Interaction', and 'Working conditions' represented burdens. The dimensions 'Professional environment' and 'Private environment' included characteristics of different coping strategies.

**Table 1:** Vignette universe.

Dimensions	Characteristics				
	1	2	3	4	Total
COVID-19 infection	fear of self-infection	fear of transmission (Ref.)			2
Interaction	Experienced loneliness	social contact (Ref.)			2
Working conditions	Compliance with hygiene and protection requirements	Additional nursing work	Additional organizational effort	staff shortage (Ref.)	4
Professional environment	collegiality	Streamline workflows	Bending guidelines (Ref.)		3
Private environment	Family members & friends	Harmful consumer behaviour (Ref.)			2
Vignette universe					96

*Notes*: Ref. = reference category.

'Social contact' referred to the circumstance in which the geriatric care nurses were unable to meet the social needs of the residents. The 'additional organisational effort' was described in the vignettes as burdensome contact with the residents' relatives.

Following the methodology outlined by Atzmüller und Steiner (2017), all 96 vignettes were randomly divided into 16 sets, each containing six vignettes. There were no illogical or implausible cases. In addition to the vignettes, an introductory vignette was formulated that included background information on the fictitious geriatric care nurse. The introductory vignette was presented to each participant alongside a short informational text before the six vignettes. Each participant received one of the 16 vignette sets at random. In the context of the presentation format of the vignettes, it should be mentioned that the burdens and coping strategies were separated by a paragraph and that the characteristics were emphasised using a bold font.

Participants were asked to indicate the extent to which they could relate to the stressful situation and the reaction of the fictitious geriatric care nurse. They evaluated the vignettes using a 10-point Likert scale ranging from 1 (“I can’t relate at all”) to 10 (“I can completely relate”).

The short informational text, the introductory vignette, and the randomised vignette set were presented together on a single screen page.

### 2.3 Statistical analyses

The data were processed with IBM SPSS Statistics (Version 29.0). Missing values were determined using listwise detection. Descriptive statistics were calculated to determine the statistical metrics of the variables of the general questionnaire. The chi-square goodness of fit test (χ^2^) was used to compare the observed with the expected number of participating nursing homes per federal state.

The dependent variable ‘vignette judgement’ was analysed applying a multilevel regression analysis. Derived from the null model, the intraclass correlation coefficients (ICC) were determined to establish whether there is sufficient explanatory variance at level 2. The multinomial scaled independent variables were dummy coded and analysed in a random intercept level 1 model. In a random intercept level 2 model, selected variables were divided into thematic blocks according to the research question (Supplementary Material, Table S2) and successively included in the analysis. Multilevel model assumptions were tested accordingly. The ICC and the log-likelihood (−2LL) were specified to determine the model quality. Pseudo-R^2^ was utilized for the analysis of level 1 and level 2 variance, as well as for the reduction of the total residual variance (Eid et al., 2017).

### 2.4 Ethical approval and informed consent

The study received ethical clearance from the Ethics Committee of the German Society for Nursing Science (No. 23–017) in July 2023. The participants were informed about the study in advance and were able to participate voluntarily after agreeing to the data and privacy policy. The internet protocol addresses of the participating end devices were not recorded and stored. The questionnaire contained variables that explicitly listed burdens in the context of the COVID-19 pandemic. Information on psychological counselling centres was provided at the end of the survey.

### 2.5 Data availability statement

At the end of the project, the data set will be made available to the public via the *GESIS - Leibniz-Institut für Sozialwissenschaften* online research database.

## 3 Results

A total of 260 participants accessed the survey; 192 participants completed it. Four participants had less than two years of professional experience and were excluded from the study. After exclusion, the final sample was decreased to *n*= 188. A χ^2^ test indicated a statistically-significant difference (χ^2^ = 39.802, *df* [degrees of freedom] = 15, *p* [probability value] < 0.001) between the observed and expected distribution of nursing homes in the federal states. Looking at the individual residuals, the largest difference was in North Rhine-Westphalia. Apart from this, the distribution was comparable. The participants estimated the average number of nursing home beds in their facility to 97.37 (standard deviation [SD] = 46, min = 14, max = 346). Descriptive statistics of the sample and of the COVID-19 related variables are summarised in Table 2 (part A).

**Table 2:** Sample characteristics and COVID-19-related variables.

A. Characteristic		n	%
Gender	Male	50	26.6
	Female	135	71.8
	Non-binary	1	0.5
Age in years	20 – 25	3	1.6
	26 – 35	26	13.8
	36 – 45	60	31.9
	46 – 55	52	27.7
	> 55	45	23.9
Operator of facility	Public	43	22.9
	Private	43	22.9
	Ecclesiastical	78	41.5
	Other	22	11.7
Highest qualification	Magister/Master	17	9.0
	Bachelor	40	21.3
	3-year training program	100	53.2
	2-year training program	7	3.7
	1-year training program	3	1.6
	Other	19	10.1
Work experience in years	2 – 5	15	8.0
	6 – 10	21	11.2
	11 – 15	22	11.7
	16 – 20	35	18.6
	> 20	94	50.0
Employment	Full-time	159	84.6
	Part-time	27	14.4
	Other	1	0.5
Active in nursing care	Yes	106	56.7
	No	81	43.1
Predominant care for residents with dementia	Yes	64	34.0
	About the same as non-dementia	101	53.7
	No	21	11.2
Living conditions	Caring for one or more children under the age of 18	73	35.4
	Caring for one or more relatives	41	19.9
	Chronically ill person(s) in own household	34	16.5
	Own chronic illness	58	28.2
**B. COVID-19 related Variables**			
Extra Work	Yes	169	89.9
	No	16	8.5
Infected with COVID-19	Nobody	1	0.2
	Residents	182	27.5
	Colleagues	182	27.5
	Their relatives	156	23.6
	Participants themselves	141	21.3
Persistent symptoms after COVID-19-infection	Yes	61	32.4
	No	80	42.6
Deaths in the personal environment as result of a COVID-19-infection	Yes	134	71.3
	There were no deaths	50	26.6
Death as result of COVID-19-infection	Residents	132	86.3
	Colleagues	10	6.5
	Their relatives	9	5.9

*Notes*: Participants (total) = 188; *n* = absolute frequency; % = relative frequency; Magister = university degree in German-speaking countries that was awarded before the Bologna Process and is equivalent to a master’s degree (European Qualifications Framework, EQF level 7); Personal environment = close personal contacts such as family members, colleagues, or residents.

### 3.1 Burden and coping strategies

Table 2 (part B) shows the descriptive frequency of the variables associated with the COVID-19 pandemic. The participants who reported residents infected with COVID-19 estimated that, on average, 54 residents were infected one or more times during the entire period of the pandemic (SD = 45, min = 0, max = 450).

**Fig. 1:**
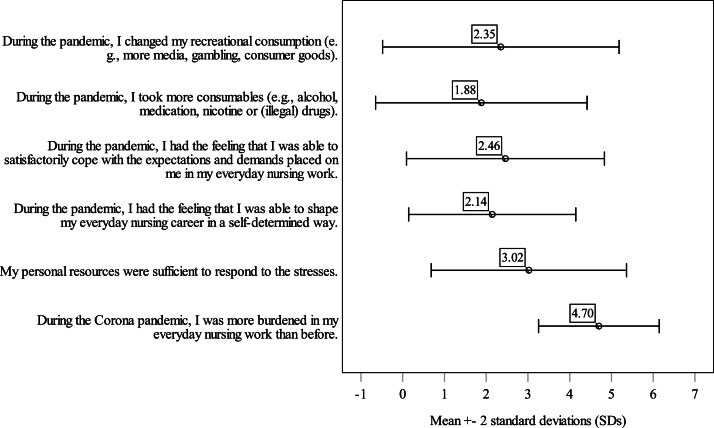
Distribution of COVID-19-related rating variables (Mean ± SD, standard deviation).

Fig. 1 depicts variables that are related to burdens, coping strategies, and professional identity (for details, see Supplementary Material, Table S3). Participants largely confirmed that they felt more burdened in their daily nursing work during the COVID-19 pandemic compared to before. In contrast, their responses indicated uncertainty about whether their personal resources were sufficient to manage these increased demands. There was a general tendency to disagree with the notion that they could shape their workday autonomously, suggesting a reduced sense of control. Coping with expectations and demands was perceived as challenging, though not overwhelmingly so. About lifestyle-related coping strategies, most participants reported little to no change in their recreational consumption patterns, and increased use of stimulants or consumables was largely rejected.

### 3.2 Vignette analysis

The testing of the requirements for the multilevel analysis revealed that there was no multicollinearity of the level 1 variables. A few correlations were identified for the level 2 variables. The level 1 and 2 residuals were approximately normally distributed. There were no noteworthy outliers or extreme values. The modified Breusch-Pagan test for heteroscedasticity revealed that the assumption of homoscedasticity was violated regarding the level 1 residuals but not for the level 2 residuals.

The a priori targeted distribution of 12 participants per vignette set was achieved in a total of seven of the 16 sets. The lowest number of vignette judgements was in the 16th set, with eight participants. The highest number of participants per set was 17. As can be seen in Supplementary Material, Table S4, the total number of vignette judgements submitted was 1119. A total of nine vignette judgements were missing. The 10-point Likert scale was used in its entire range. The mean value of all vignette judgements was 6.55 (SD = 3.11, min = 1, max = 10). About a quarter of the participants rated a judgement of 10 (“I can completely relate”). Table 3 summarises the average vignette judgements for each vignette characteristic.

**Table 3:** Average vignette judgement per characteristic.

Dimensions	Characteristics	(Valid) Vignette judgements (missing)	Mean (SD)
COVID-19 infection	Fear of self-infection	558 (4)	6.71 (2.98)
COVID-19 infection	Fear of transmission	561 (5)	6.38 (3.23)
Interaction	Experienced loneliness	552 (4)	6.53 (3.13)
Interaction	Social contact	567 (5)	6.56 (3.10)
Working conditions	Compliance with hygiene and protection requirements	287 (1)	6.63 (3.21)
Working conditions	Additional nursing work	286 (1)	6.61 (2.95)
Working conditions	Additional organizational effort	266 (3)	6.57 (3.15)
Working conditions	Staff shortage	280 (4)	6.38 (3.15)
Professional environment	Collegiality	370 (4)	6.66 (3.15)
Professional environment	Streamline workflows	377 (1)	6.60 (3.02)
Professional environment	Bending guidelines	372 (4)	6.38 (3.17)
Private environment	Family members & friends	560 (7)	7.92 (2.36)
Private environment	Harmful consumer behaviour	559 (2)	5.17 (3.17)

*Notes*: SD, standard deviation.

### 3.3 Multilevel regression analysis

Table 4 presents the results of the null model and the random intercept model at levels 1 and 2. Table 4 contains statistical values of all the level 1 model variables and all the level 2 model variables with *p*-values less than 0.05. Full details of the multilevel model are listed in Supplementary Material, Table S5. Based on the null model, the ICC is 0.410, suggesting the appropriateness of a hierarchical model.

**Table 4:** Random intercept model (vignette and participant level).

	Null model		RI level 1 model		RI level 2 model	
	β [95 % CI]	*p*-value	β [95 % CI]	*p*-value	β [95 % CI]	*p*-value
Intercept	6.534 [6.214, 6.854]	< 0.001	4.721 [4.255, 5.186]	< 0.001	5.582 [2.264, 8.900]	0.001
Fear of self-infection			0.231 [−0.011, 0.473]	0.062	0.104 [−0.186, 0.394]	0.480
Fear of transmission (Ref.)						
Compliance with hygiene and protection requirements			0.063 [−0.289, 0.415]	0.725	0.019 [−0.387, 0.426]	0.926
Additional nursing work			0.264 [−0.082, 0.610]	0.135	0.096 [−0.328, 0.520]	0.656
Additional organisational effort^1^			0.049 [−0.308, 0.407]	0.786	−0.046 [−0.479, 0.388]	0.837
Staff shortage (Ref.)						
Experienced loneliness			0.044 [−0.204, 0.292]	0.729	0.034 [−0.261, 0.329]	0.822
Social contact (Ref.)						
Collegiality			0.465 [0.150, 0.781]	0.004	0.385 [0.002, 0.767]	0.049
Streamline workflows			0.357 [0.029, 0.685]	0.033	0.327 [−0.062, 0.716]	0.100
Bending guidelines (Ref.)			.	.	.	.
Family members & friends			2.606 [2.359, 2.853]	< 0.001	2.791 [2.494, 3.088]	< 0.001
Harmful consumer behaviour (Ref.)						
Recreational consumption^a^					0.296 [0.039, 0.552]	0.024
Residents					−0.009 [−0.017, −0.002]	0.019
Higher Load during COVID-19 pandemic^a^					1.464 [0.587, 2.340]	0.001
Professional experience 2 – 5 years					1.764 [0.494, 3.033]	0.007
Professional experience 6 – 10 years					1.100 [0.017, 2.183]	0.047
Professional experience > 20 years (Ref.)						
ICC	0.410		0.500		0.391	
−2LL	5436.191		5061.917		3354.653	
Total R^2^			0.197		0.364	
Level 1 R^2^			0.319			
Level 2 R^2^					3.393	

*Notes*:.

a grand-mean centred; β = beta regression coefficients; For participant characteristics, only *p* < 0.05 reported; CI, confidence interval; ICC, intraclass correlation coefficient; Ref., reference category; RI, Random Intercept; −2LL, Log-Likelihood.

#### 3.3.1 Random intercept level 1 model

In the random intercept level 1 model, vignette characteristics were included as fixed factors, with nominal variables analysed relative to predefined reference categories. The inclusion of five variables explained 31.9 % of the level 1 residual variance and approximately 20 % of the total residual variance.

The burden-related vignette variables did not show a statistically-significant influence on participants' judgement behaviour, while certain coping strategies did: "collegiality" was rated higher than the reference category "bending guidelines." However, when compared to "streamline workflows," "collegiality" showed no significant difference. Interestingly, "streamline workflows" itself had a significant positive influence when compared to "bending guidelines." In the private environment dimension, the strategy of seeking support from "family members and friends" was rated substantially higher than the maladaptive strategy of "harmful consumer behaviour."

#### 3.3.2 Random-Intercept level 2 model

By adding the fixed level 2 variables to the random intercept level 2 model, 39.3 % of level 2 residual variance was explained. In total, 36.4 % of the total residual variance can be explained.

In the random intercept level 2 model, the burden-related vignette variables – consistent with the level 1 model – did not have a statistically-significant effect on participants' judgement behaviour. Among the coping strategies, both “collegiality” and “family members and friends” continued to show a significant positive influence. In contrast, “streamline workflows” did not reach statistical significance compared to the reference category “bending guidelines”.

In the random intercept level 2 model, three coping-related variables were included: changes in recreational consumption, use of consumables, and personal resources for managing stress. Among these, only “recreational consumption” showed a statistically-significant influence on judgement behaviour, while “resources” and “consumables” did not.

Regarding burdens, six socio-demographic variables were included. A higher estimated number of residents with COVID-19 one or more times was associated with lower ratings, whereas a higher perceived workload during the pandemic had a positive effect on judgement. Other factors – such as working overtime, exposure to COVID-19-related deaths, and long-term COVID symptoms – did not significantly affect participants’ judgements. The sum index variable “personal circumstances”, counting how many of the presented personal circumstances apply, showed no significant differences across levels compared to the reference category (“4 circumstances”).

In the domain of professional identity, neither “autonomy” nor “competence” significantly influenced judgement behaviour.

Finally, two additional factors were tested: professional experience and involvement in direct care. Participants with 2–5 years of experience rated vignettes significantly higher than those with over 20 years of experience. Similarly, those with 6–10 years of experience rated higher. However, experience levels of 11–15 years and 16–20 years were not statistically-significant. Whether participants were directly involved in nursing care had no significant effect on vignette judgement.

## 4 Discussion

We conducted a factorial survey to identify the burdens and coping strategies experienced as relevant by the geriatric care nurses in German nursing homes during the COVID-19 pandemic, considering their professional identity. The 188 participants were more stressed during the COVID-19 pandemic in their everyday nursing work than before. The additional burden was significantly related to their judgement behaviour. However, the multi-level analysis indicated that none of the burden characteristics defined in the vignettes had a statistically-significant influence on the judgement behaviour of the participants. In the private environment, closeness to family and friends proved to be a more effective coping strategy than relaxation in an increased consumption of stimulants. In the professional environment, seeking support from team colleagues proved to be more effective than bending the COVID-19-specific guidelines. The autonomous and competent organization of everyday nursing work was restricted during the COVID-19 pandemic but was not related to the assessment of burdens and coping strategies in the vignettes.

### 4.1 COVID-19 pandemic-related burdens and coping strategies

None of the burden characteristics had a statistically-significant influence on participants' judgement behaviour. It should be noted here that by setting a reference category, not all burden characteristics were compared with each other. A further methodological explanation could be the fact that the burdens and coping reactions of the fictitious geriatric care nurse were assessed together in the vignettes. Consequently, the coping strategies described may have played a greater role in the participants' judgement than the burdens. The participants almost entirely agreed with the statement that they were under greater burden during the pandemic than before. Looking at the pre-pandemic situation in German geriatric care, geriatric care nurses were already exposed to a high level of stress (Jacobs et al., 2020). Although many of these factors, such as the number of staff absences and working overtime, were exacerbated by the pandemic (Piechotta-Henze et al., 2022), they were already familiar conditions. The additional burden factors added by the pandemic, such as the implementation of hygiene and protective requirements, may have led to such an extraordinary state of general burden (Cockshott et al., 2024) that none of the individual burden factors stood out. If the burden factors are not considered individually but as an entity in which the factors interact with each other, the result would be a cumulative experience of burden. It is therefore an additive value that includes the experience and assessment of chronic stressors in different areas of life over a longer period (Haight et al., 2023). In the case of the COVID-19 pandemic, geriatric care nurses were faced with challenges that varied in severity and frequency over a 3-year period, which may have led to the experienced state of cumulative burden. Haight et al. (2023) demonstrated that high cumulative stress and daily stressors increased the likelihood of physical health problems. For some of the participants who answered the open questions at the end of the questionnaire, this extraordinary state of burden led them to internally distance themselves from the burdening factors. This self-protection mechanism could have led them to not evaluating the burdens described in the vignettes in a particular way. In addition to the internal factors that may have contributed to the fact that no differences were found between the stress levels, Giri et al. (2021) emphasised that not every nursing home was affected to the same extent and by the same requirements. This individuality of the burden factors at the facility level could also explain why there were no overarching burden factors that stood out as particularly prominent.

We found that closeness to family and friends as opposed to increased consumption of stimulants and seeking support from team colleagues, as opposed to bending guidelines as a reaction to the burden, were more strongly understood by the participants. If we look at the reference categories in this case, it becomes clear that both represent socially-undesirable behaviour and could have influenced the judgements to this effect. Closeness to family and friends, as well as support from colleagues, can be categorised in the family of coping ‘support seeking’, according to the Skinner et al. (2003) coping taxonomy, which highlights the use of social resources as a function in the adaptive process. This result is consistent with previous findings that carers have frequently sought support in interpersonal relationships during the pandemic (Dürr et al., 2022; Müller et al., 2021), despite the restrictive pandemic regulations on social distancing. Recent research has also demonstrated the positive influence of seeking social support as a coping strategy on the mental health of carers (Labrague, 2021; Labrague and Los Santos, 2020). As also postulated in the core of the ‘social buffering hypothesis’ (Cohen and Wills, 1985), social support-seeking appears to play a fundamental role in mental health, which was affected during the COVID-19 pandemic.

In contrast, the two coping strategies ‘harmful consumer behaviour’ and ‘bending guidelines’ have not proved to be successful from the perspective of the participants. The rejection of both coping strategies in favour of seeking social support could also be related to the negative health, social, and legal consequences of these actions. For example, violating the COVID-19 regulations could had legal consequences (German Infection Protection Act, §§73–75 IfSG). Bending rules and guidelines, such as not wearing a face mask appropriately, could have been associated with an increased fear of infection for many geriatric care nurses (Blanco-Donoso et al., 2021; Podgorica et al., 2022). The fear of infecting themselves or others with COVID-19 was also present in the free-text responses. To cope with this fear, the participants also stated that they sometimes reduced their social contacts. Bending the guidelines also potentially jeopardised their own jobs at a time when many people were losing their jobs or businesses. Therefore, in addition to legal and social reasons, socio-economic considerations may have played an important role. In contrast to the results of the factorial survey, the study situation on the increased consumption of stimulants during the COVID-19 pandemic is less clear. For example, Xu et al. (2021) found in a literature review that drinking and online gaming behaviours increased during the COVID-19 pandemic, but Perkhofer et al. (2021) identified only a moderate increase in harmful consumption behaviour, limited to those younger than 35. Preliminary analyses of our study’s free-text responses also indicated that the consumption of stimulants played a subordinate role compared to the search for social support. Some participants even explicitly stated in their comments that the consumption of stimulants was not essential to them. This could be explained by the fact that many participants were at the end of their tether because of the additional workload and staff shortages. The harmful consumption of stimulants would have tended to exacerbate this condition, which could be explain why the participants did not resort to this maladaptive coping strategy.

### 4.2 Professional identity

In the context of the participants' professional identity, it became clear that the experience of autonomy and competence among geriatric care nurses was affected by the pandemic (Begerow and Gaidys, 2022; Flatscher-Thöni et al., 2022). The experience of autonomy and competence was not varied in the vignettes as independent dimensions but was integrated into the introductory vignette. It is, therefore, not possible to record the influence of these two factors on the judgement behaviour of the participants at the vignette level. As the experience of competence and autonomy is also of central importance for coping with burden (Skinner et al., 2003), these were included in the general questionnaire section and as level 2 variables in the multilevel analysis. Although the participants considered their personal resources to be sufficient on average, they did not feel that they were able to cope satisfactorily with the expectations and demands placed on them in their day-to-day work. This suggests that although they had sufficient resources to cope with the crisis, the loss of autonomy they experienced made it difficult to cope competently. This result is in line with the findings of Philippa et al. (2021), who found that autonomous and competent action was fundamental to effective and successful care provision. The fact that autonomous action was limited during the COVID-19 pandemic was not least because the frequently-changing hygiene and protection requirements gave little scope for autonomous but also adaptively competent problem-solving behaviour (Begerow and Gaidys, 2022; Diehl et al., 2022). As a result, some of the participants reported that they had the feeling that their day-to-day care was being controlled by others. Freytag et al. (2021) went even further and found that nurses reported a feeling of coercion when carrying out the measures. The pandemic crisis resulted in geriatric carer nurses experiencing a regression to functional care, in which the autonomous and competent actions of the individual receive little attention (Begerow and Gaidys, 2022). In line with self-determination theory (Deci and Ryan, 2000) and the study results, the experience of autonomy and competence took a back seat during the COVID-19 pandemic due to the external regulation of professional carers' scope of action and consequently played a subordinate role in coping with crisis-related burdens.

At this point, well-known discussions on moral distress (Johnson et al., 2017), as well as the causes of burnout, post-traumatic stress disorder, anxiety, and depression follow, as they are also described for the pandemic (Ernst et al., 2020). As defined, it was not the acute stress alone that causes this psychological, somatic, and psychosomatic consequences (Glaser et al., 2015), but the inner state of tension experienced by the caregivers must be taken into account. This inner tension, in turn, results from various factors; e.g., part of the professional identity of geriatric carer nurses is to understand themselves as care workers and, therefore, to want to realize an ideal of good care that often cannot be realized in everyday life. Tensions are also triggered by unsuitable or even harmful organizational conditions. However, positive organizational measures to relieve the burden on caregivers can also be identified for the pandemic. These include the adapting of workflows (Begerow and Gaidys, 2022; Diehl et al., 2022; Flatscher-Thöni et al., 2022), convening of COVID-19 pandemic teams for better organization and information dissemination, or finding creative solutions to reduce the experience of stress, most common for the lack of social contacts (Eich-Krohm et al., 2022; Sander et al., 2023).

### 4.3 Limitations

The cross-sectional design limits the ability to interpret the findings in a causal manner (Wang and Cheng, 2020). Since the data were collected retrospectively at a point in time after the COVID-19 pandemic, it is not possible to determine whether the perceived impact of burdens and coping strategies might have been evaluated differently by participants during the pandemic itself.

By design, factorial surveys capture hypothetical decisions, beliefs, and intentions, which, although useful for isolating specific variables, may not fully capture the emotional complexity and contextual nuance of real-life decision-making. Participants' responses to these scenarios might differ from their actual behaviours in real-world situations, potentially limiting the external validity of the findings. The retrospective survey may not fully reflect the dynamic nature of coping mechanisms during a crisis like the COVID-19 pandemic. Additionally, we focused primarily on individual-level coping strategies and did not examine organizational factors that may influence individuals’ stress and coping, such as staffing levels, availability of personal protective equipment, institutional communication, training, and support structures.

Building upon a total of *N*= 188 participants, the a priori determined sample size of *n*= 192 was basically met. However, the intended 12 judgements per vignette set were achieved only in seven of 16 sets. Despite this, the dataset included just nine missing values and 1119 valid vignette judgements, suggesting good data quality. This is supported by participant survey feedback, with the vast majority finding the vignettes generally understandable, although around 20 % felt they did not realistically depict the COVID-19 context. The limited variation in burden ratings may result from participants providing only one judgement per vignette, possibly focusing more on the fictitious nurse’s reactions than on the burdens themselves. In addition, as the vignette universe included harmful consumption as a coping strategy, participants may have been inclined to favour more socially-acceptable responses, such as seeking social support, thereby potentially biasing judgement behaviour.

Statistically, the violation of the homoscedasticity assumption at level 1 may bias parameter estimates. A correlation above 0.5 and p < 0.05 served as the multicollinearity threshold, though undetected correlations below this level may still affect regression accuracy. Further, subgroup sizes for level 2 variables varied, and categories with fewer judgements were avoided as reference groups where possible but not in every case.

While the findings may be cautiously generalized across countries facing similar challenges during the COVID-19 pandemic, specific results should be interpreted within national, cultural, and legal contexts.

### 4.4 Recommendations for practice and future research

We have underscored the critical role of social connection and peer support in coping with the unique challenges during crisis. Despite financial constraints, nursing home managers and healthcare administrators might consider focusing on promoting a supportive work environment by encouraging open communication, team reflection, coaching, peer support groups, or regular check-ins to prepare for future crisis. Institutions could be equipped to offer such services in timely and flexible ways, such as web-based formats. Institutional preventive measures to encourage professional autonomy and acknowledge emotional work demands could involve the establishment of retreat spaces for breaks, the provision of sufficient personal protective equipment, or meditation and mindfulness training. Clear, consistent communication paired with flexible expectations may help reduce reliance on maladaptive coping strategies, such as bending rules or harmful consumption. These findings may suggest that effective coping is not only an individual matter but one shaped significantly by the organizational context. Interventions designed to promote psychological well-being may, therefore, operate at both personal and systemic levels, addressing working conditions, recognition, and social and emotional support simultaneously.

Next to examining the effectiveness of the above-mentioned possibly preventive measures in practice, future researchers using factorial survey methodology could separately assess burdens and coping strategies, while incorporating elements, such as professional identity, into the vignettes. A more comprehensive analysis that includes these organizational dynamics would provide important insight into the systemic factors contributing to nurses’ well-being during crisis situations. Longitudinal designs would also be beneficial to track how coping strategies and burden perceptions evolve over time. This would provide further evidence of temporal dynamics of psychological adaptation and resilience, particularly among nurses in long-term care. Qualitative or mixed-methods approaches could complement the vignette-based findings by offering a richer understanding of individual meaning-making processes and contextual influence. Finally, broadening the scope to include other healthcare professions and systems to assess the cultural and institutional variability of coping mechanisms would enhance and inform more tailored interventions for different national or occupational contexts.

## 5 Conclusions

The burden on geriatric care nurses in Germany was greater than before the COVID-19 pandemic. None of the burden characteristics varied in the vignettes had a statistically-significant influence on the participants' judgement behaviour. Closeness to family and friends proved particularly beneficial compared to harmful consumer behaviour and seeking support from team colleagues compared to bending guidelines. As a result, bending of guidelines and harmful consumer behaviour were not adequate coping strategies for geriatric care nurses. Although their own perception of autonomy and competence as elements of their professional identity were impaired during the pandemic, this did not affect their assessment of the coping strategies used to deal with burdens.

## Funding sources

This work was supported by the Federal Ministry of Education and Research [grant number: 01UP2223]. The sponsor was not involved in study design, writing of the report, or any other decisions regarding the research process and publication.

## CRediT authorship contribution statement

**Christopher Huken:** Writing – review & editing, Writing – original draft, Methodology, Investigation, Formal analysis, Data curation, Conceptualization. **Patrick Kutschar:** Writing – review & editing, Methodology, Investigation, Formal analysis, Conceptualization. **Martin W. Schnell:** Writing – review & editing, Supervision, Project administration, Methodology, Investigation, Funding acquisition, Conceptualization. **Christine Dunger:** Writing – review & editing, Supervision, Project administration, Methodology, Investigation, Funding acquisition, Conceptualization.

## Declaration of competing interest

The authors declare that they have no known competing financial interests or personal relationships that could have appeared to influence the work reported in this paper.

## Appendix Supplementary materials

Image, application 1
