# Supplementary material for: Burdens and coping strategies among geriatric care nurses during COVID-19: A factorial survey
Source: Int J Nurs Stud Adv. 2025 Jul 19;9:100386. doi: 10.1016/j.ijnsa.2025.100386 (PMC12314172; doi:10.1016/j.ijnsa.2025.100386)
Supplement: Supplementary file 1 [file mmc1.docx]

# Supplementary Material

This additional document provides supplementary material to the publication. Links can be found in the publication. This is material on the questionnaire and further statistical results.

**Table S1**

Sociodemographic and COVID-19 related variables

| **Variable** | **Characteristics** |
| --- | --- |
| 1. Which gender do you feel you belong to?^1^ | Male, Female, Non-binary |
| 2. How old are you?^1^ | < 20, 20 – 25, 26 – 35, 36 – 45, 46 – 55, > 55 |
| 3. In which federal state do you work?^1^ | Baden-Württemberg, Bavaria, Berlin, Brandenburg, Bremen, Hamburg, Hesse, Mecklenburg Western Pomerania, Lower Saxony, Northrhine-Westphalia, Rhineland Palatinate, Saarland, Saxony-Anhalt, Saxony, Schleswig-Holstein, Thuringia |
| 4. The provider of your institution is…^1^ | Public, Private, Ecclesiastical, Other |
| 5. How many inpatient care places are there in your facility? Please enter 0 if you do not wish to provide any information. | Numerical input |
| 6. Please indicate your highest qualification.^1^ | University (Magister/Master), University (Bachelor), Graduation after 3 years of vocational training, Graduation after 2 years of vocational training, 1 year of vocational training or shorter, Other |
| 7. How much experience in nursing do you have after completing your vocational training?^1^ | Less than 2 years, 2 – 5 years, 6 – 10 years, 11 – 15 years, 16 – 20 years, More than 20 years |
| 8. Do you work in the direct nursing care of the residents?^1^ | Yes, No |
| 9. How long did you work during the pandemic?^1^ | Full-time, Part-time, Other/changing job shares |
| 9.1 If Part-time^1^ | More than 50 % of jobs, Exactly 50 % job share, Less than 50 % of the workforce |
| 10. Are you affected by overtime, in the sense of working overtime, due to the pandemic?^1^ | Yes, No |
| 11. Did you mainly care for residents with dementia during the pandemic?^1^ | Yes, About the same amount, No |
| 12. Who in your immediate environment was infected with COVID-19 during the pandemic?^2^ | Nobody, Residents, Colleagues, Your relatives, Autonomy |
| 12.1 When Residents: If you think back over the entire period of the coronavirus pandemic (2020 – 2023), how many residents have you looked after who have contracted COVID-19 one or more times? | Numerical input |
| 12.2 When Autonomy: Do you still suffer from symptoms associated with COVID-19 infection after the COVID-19 infection?^1^ | Yes, No |
| 13. Have there been any deaths in your environment as a result of a COVID-19 infection?^1^ | Yes, There were no deaths |
| 13.1 When Yes: Who died as a result of the COVID-19 infection?^2^ | Residents, Colleagues, Own relatives, Not specified |
| 14. Do one or more of the following statements apply to your personal circumstances?^2^ | Care of one or more children under the age of 18, Caring for one or more relatives, Chronically ill person(s) in their own household, Own chronic illness |
| 15. Matrix-Question: To what extent do you agree with the following statements?  15.1 During the Corona pandemic, I was more burdened in my everyday nursing work than before.  15.2 My personal resources were sufficient to respond to the stresses.  15.3 During the pandemic, I had the feeling that I was able to shape my everyday nursing career in a self-determined way.  15.4 During the pandemic, I had the feeling that I was able to satisfactorily cope with the expectations and demands placed on me in my everyday nursing work.  15.5 During the pandemic, I took more consumables (e.g. alcohol, medication, nicotine or (illegal) drugs).  15.6 During the pandemic, I changed my recreational consumption (e.g. more media, gambling, consumer goods). | 5-point Likert-Scale (Strongly disagree, Rather disagree, Neutral, Tend to agree, Totally agree) |
| 16. Have you experienced increased media coverage of your profession during the Corona pandemic?^1^ | Yes, No |
| 17. Was your professional situation in the care home during the coronavirus pandemic portrayed appropriately in the media?^1^ | Yes, No |
| 18. Matrix-Question: Which media did you consume the most during the Corona pandemic?  18.1 Social media (e.g. Facebook, Twitter)  18.2 Messenger services (e.g. Telegram)  18.3 Podcasts  18.4 TV Programs  18.5 Radio broadcasts  18.6 (Online-)Periodicals | 5-point Likert-Scale (Never, Seldom, Sometimes, Frequently, All the time) |
| 19. Matrix-Question: How strongly do you agree with the following statements?  19.1 Overall, the vignettes were understandable.  19.2 The vignettes realistically depict the Corona pandemic. | 5-point Likert-Scale (Strongly disagree, Rather disagree, Neutral, Tend to agree, Totally agree) |
| 20. In the following free text field, you can list experienced stress factors that were particularly relevant to you during the pandemic and may not have been recorded by the study. |  |
| 21. In the following free text field, you can list coping strategies that were particularly helpful for you during the pandemic and that may not have been recorded by this study. |  |
| 22. In the following free text field, you have the opportunity to note any additions or comments. |  |

*Notes*: ^1^Single response; ^2^Multiple response; Magister = university degree in German-speaking countries that was awarded before the Bologna Process and is equivalent to a Master's degree (European Qualifications Framework, EQF level 7).

**Table S2**

Level 2 variables divided into thematic blocks

| **Block** | **Variables** |
| --- | --- |
| 1. Coping-Strategies | 15.2, 15.5, 15.6 |
| 2. Burdens | 10, 12.1, 12.2, 13, 14, 15.1 |
| 3. Professional Identity | 15.3, 15.4 |
| 4. General Variables | 7, 8 |

*Notes:* The variable numbers represent the numbering from Table S1.

**Table S3**

Matrix variables

| **Item** | **n (missings)** | **Mean (SD)** |
| --- | --- | --- |
| Higher load during COVID-19 pandemic | 187 (1) | 4.70 (0.72) |
| Resources | 183 (5) | 3.03 (1.16) |
| Autonomy | 184 (4) | 2.15 (1.01) |
| Competence | 185 (3) | 2.49 (1.19) |
| Consumables^1^ | 185 (3) | 1.89 (1.26) |
| Recreational consumption^2^ | 186 (2) | 2.34 (1.42) |

*Notes*: ^1^ e.g. alcohol, medication, nicotine or (illegal) drugs. ^2^ e.g. more media, gambling, consumer goods; n = participants; SD, standard deviation.

**Table S4**

Vignette judgments

| **Vignette Set Nr.** | **Participants** | **(Valid) vignette judgements** | | **Valid frequency** | **Missing judgments per set** |
| --- | --- | --- | --- | --- | --- |
| 1 | 11 | | 66 | 5.9 | 0 |
| 2 | 13 | | 78 | 6.9 | 0 |
| 3 | 9 | | 54 | 4.8 | 0 |
| 4 | 8 | | 48 | 4.2 | 0 |
| 5 | 15 | | 87 | 7.7 | 3 |
| 6 | 11 | | 64 | 5.7 | 2 |
| 7 | 9 | | 54 | 4.8 | 0 |
| 8 | 13 | | 78 | 6.9 | 0 |
| 9 | 11 | | 63 | 5.6 | 3 |
| 10 | 17 | | 102 | 9.1 | 0 |
| 11 | 12 | | 72 | 6.4 | 0 |
| 12 | 14 | | 84 | 7.5 | 0 |
| 13 | 17 | | 102 | 9.1 | 0 |
| 14 | 11 | | 65 | 5.8 | 1 |
| 15 | 9 | | 54 | 4.8 | 0 |
| 16 | 8 | | 48 | 4.2 | 0 |
| **Total** | **188** | | **1,119** | **100** | **9** |

**Table S5**

Random intercept model (vignette and respondent level)

|  | **Null Model** | | **RI Level 1 Model** | | **RI Level 2 Model** | |
| --- | --- | --- | --- | --- | --- | --- |
|  | **β [95% CI]** | **p-value** | **β [95% CI]** | **p-value** | **β [95% CI]** | **p-value** |
| Intercept | 6.534 [6.214, 6.854] | < 0.001 | 4.721 [4.255, 5.186] | < 0.001 | 5.582 [2.264, 8.900] | 0.001 |
| Fear of self-infection |  |  | 0.231 [-0.011, 0.473] | 0.062 | 0.104 [-0.186, 0.394] | 0.480 |
| Fear of transmission (Ref.) |  |  |  |  |  |  |
| Compliance with hygiene and protection requirements |  |  | 0.063 [-0.289, 0.415] | 0.725 | 0.019 [-0.387, 0.426] | 0.926 |
| Additional nursing work |  |  | 0.264 [-0.082, 0.610] | 0.135 | 0.096 [-0.328, 0.520] | 0.656 |
| Additional organisational effort^1^ |  |  | 0.049 [-0.308, 0.407] | 0.786 | -0.046 [-0.479, 0.388] | 0.837 |
| Staff shortage (Ref.) |  |  |  |  |  |  |
| Experienced loneliness |  |  | 0.044 [-0.204, 0.292] | 0.729 | 0.034 [-0.261, 0.329] | 0.822 |
| Social contact^2^ (Ref.) |  |  |  |  |  |  |
| Collegiality |  |  | 0.465 [0.150, 0.781] | 0.004 | 0.385 [0.002, 0.767] | 0.049 |
| Streamline workflows |  |  | 0.357 [0.029, 0.685] | 0.033 | 0.327 [-0.062, 0.716] | 0.100 |
| Bending guidelines (Ref.) |  |  |  |  |  |  |
| Family members & friends |  |  | 2.606 [2.359, 2.853] | < 0.001 | 2.791 [2.494, 3.088] | < 0.001 |
| Harmful consumer behaviour (Ref.) |  |  |  |  |  |  |
| Resources^a^ |  |  |  |  | -0.191 [-0.538, 0.156] | 0.277 |
| Consumables^a^ |  |  |  |  | 0.185 [-0.120, 0.490] | 0.232 |
| Recreational consumption^a^ |  |  |  |  | 0.296 [0.039, 0.552] | 0.024 |
| Working overtime: Yes |  |  |  |  | -0.108 [-1.873, 2.657] | 0.904 |
| Working overtime: No |  |  |  |  |  |  |
| Symptoms after COVID-19 Infection: Yes |  |  |  |  | -0.547 [-1.256, 0.161] | 0.129 |
| Symptoms after COVID-19 Infection: No |  |  |  |  |  |  |
| COVID-19 deaths: Yes |  |  |  |  | 0.312 [-0.485, 1.109] | 0.440 |
| COVID-19 deaths: No |  |  |  |  |  |  |
| Personal circumstances: 0 |  |  |  |  | -0.869 [-4.127, 2.388] | 0.598 |
| Personal circumstances: 1 |  |  |  |  | -0.798 [-3.981, 2.385] | 0.621 |
| Personal circumstances: 2 |  |  |  |  | -1.149 [-4.357, 2.058] | 0.480 |
| Personal circumstances: 3 |  |  |  |  | -0.733 [-4.085, 2.620] | 0.666 |
| Personal circumstances: 4 |  |  |  |  |  |  |
| Residents |  |  |  |  | -0.009 [-0.017, -0.002] | 0.019 |
| Higher Load during COVID-19 pandemic^a^ |  |  |  |  | 1.464 [0.587, 2.340] | 0.001 |
| Autonomy^a^ |  |  |  |  | -0.337 [-0.739, 0.065] | 0.099 |
| Competence^a^ |  |  |  |  | 0.051 [-0.263, 0.364] | 0.749 |
| Direct nursing care: Yes |  |  |  |  | 0.011 [-0.701, 0.723] | 0.967 |
| Direct nursing care: No |  |  |  |  |  |  |
| Professional experience 2 – 5 years |  |  |  |  | 1.764 [0.494, 3.033] | 0.007 |
| Professional experience 6 – 10 years |  |  |  |  | 1.100 [0.017, 2.183] | 0.047 |
| Professional experience 11 – 15 years |  |  |  |  | 0.853 [-0.477, 2.184] | 0.207 |
| Professional experience 16 – 20 years |  |  |  |  | 0.789 [-0.064, 1.642] | 0.070 |
| Professional experience > 20 years (Ref.) |  |  |  |  |  |  |
| ICC | 0.410 |  | 0.500 |  | 0.391 |  |
| -2LL | 5436.191 |  | 5061.917 |  | 3354.653 |  |
| Total R^2^ |  |  | 0.197 |  | 0.364 |  |
| Level 1 R^2^ |  |  | 0.319 |  |  |  |
| Level 2 R^2^ |  |  |  |  | 0.393 |  |

*Notes*: ^a^grand-mean-centered; β = beta regression coefficients; CI, 95 % confidence interval; ICC, intraclass correlation coefficient; Ref., reference category; RI, Random Intercept; -2LL, Log-Likelihood.
